# Renal fibrosis due to multiple cisplatin treatment is exacerbated by kinin B1 receptor antagonism

**DOI:** 10.1590/1414-431X2021e11353

**Published:** 2021-10-18

**Authors:** A. Budu, L.C. Freitas-Lima, A.C. de Arruda, M.S. Perilhão, J. Barrera-Chimal, R.C. Araújo, G.R. Estrela

**Affiliations:** 1 Departamento de Biofísica, Universidade Federal de São Paulo, São Paulo, SP, Brasil; 2 Disciplina de Nefrologia, Departamento de Medicina, Universidade Federal de São Paulo, São Paulo, SP, Brasil; 3 Faculdade de Educação Física, Universidade Santo Amaro, São Paulo, SP, Brasil; 4 Instituto de Investigaciones Biomédicas, Universidad Nacional Autónoma de México, Mexico City, Mexico; 5 Unidad de Investigación UNAM-INC, Instituto Nacional de Cardiología Ignacio Chávez, Mexico City, Mexico; 6 Disciplina de Hematologia e Hematoterapia, Departamento de Oncologia Clínica e Experimental, Universidade Federal de São Paulo, São Paulo, SP, Brasil

**Keywords:** Chronic kidney disease, Cisplatin nephrotoxicity, Renal fibrosis, Kinins, Kinin B1 receptor

## Abstract

Cisplatin is a widely used chemotherapeutic drug, but its side effects are a major limiting factor. Nephrotoxicity occurs in one third of patients undergoing cisplatin treatment. The acute tubular injury caused by cisplatin often leads to a defective repair process, which translates into chronic renal disorders. In this way, cisplatin affects tubular cells, and maladaptive tubules regeneration will ultimately result in tubulointerstitial fibrosis. Kinins are well known for being important peptides in the regulation of inflammatory stimuli, and kinin B1 receptor deficiency and antagonism have been shown to be beneficial against acute cisplatin nephrotoxicity. This study aimed to analyze the effects of kinin B1 receptor deletion and antagonism against repeated cisplatin-induced chronic renal dysfunction and fibrosis. Both the deletion and the antagonism of B1 receptor exacerbated cisplatin-induced chronic renal dysfunction. Moreover, the inhibition of B1 receptor increased tubular injury and tubulointerstitial fibrosis after repeated treatment with cisplatin. The balance between M1/M2 macrophage polarization plays an important role in renal fibrosis. Kinin B1 receptor antagonism had no impact on M1 markers when compared to cisplatin. However, YM1, an M2 marker and an important molecule for the wound healing process, was decreased in mice treated with kinin B1 receptor antagonist, compared to cisplatin alone. Endothelin-1 levels were also increased in mice with B1 receptor inhibition. This study showed that kinin B1 receptor inhibition exacerbated cisplatin-induced chronic renal dysfunction and fibrosis, associated with reduced YM1 M2 marker expression, thus possibly affecting the wound healing process.

## Introduction

Acute kidney injury (AKI) is associated with increased mortality and affects up to half of patients in intensive care units (1,2). One of the consequences of AKI is the increased risk of chronic kidney disease (CKD) (3,4). Acute tubular injury leads to the activation of several tissue repair mechanisms aimed at restoring renal function (3,4). This process consists of consecutive events, but if these repair mechanisms are interrupted, inefficient, or the injury stimulus persists, acute injury can progress to a chronic disorder (3,4). Several studies have shown that AKI enhances fibrogenesis, which leads to an increased risk of CKD progression (5). Tubulointerstitial fibrosis is one of the characteristics of CKD, mainly due to the excessive deposition of extracellular matrix (ECM) and the presence of collagen fibers (6).

Cisplatin is a platinum-based compound commonly used as a chemotherapeutic agent, and is very effective against a number of carcinomas, sarcomas, and lymphomas (7,8). On the other hand, dose-dependent acute nephrotoxicity is observed, which limits its administration (9). Cisplatin-induced nephrotoxicity is due to several mechanisms, including direct tubular toxicity, oxidative stress, inflammation, DNA damage, and apoptosis (7,8).

Kinins are widely known to affect the inflammatory response (10-13). Their effects are mediated by two G-protein-coupled transmembrane receptors, namely: Kinin B2 receptor (B2R), which is constitutively expressed and is responsible for most kinins effects (13), and Kinin B1 receptor (B1R), which usually has low expression levels and is highly upregulated after inflammatory stimuli (14). Our group has previously reported that the deletion and blockage of both B1R and B2R can mitigate acute cisplatin nephrotoxicity (15,16).

About 30% of patients undergoing cisplatin treatment develop AKI, which can progress to CKD (7,8). Considering that B1R deletion and antagonism can attenuate cisplatin-induced AKI, this study investigated whether B1R antagonism and deletion can prevent CKD progression after multiple doses of cisplatin.

## Material and Methods

### Animals

Male C57BL/6 and Bdkrb1 knockout (B1KO) mice weighing 23-27 g and aged 10-12 weeks were used for these experiments. The animals were obtained from the Animal Care Facility of the Universidade Federal de São Paulo (UNIFESP). All animals were housed in standard cages and had free access to water and food. All procedures were previously reviewed and approved by the internal ethical committee of the Universidade Federal de São Paulo (CEUA 3456260419).

### Experimental protocol

For the experiments evaluating B1 receptor in knockout animals, the mice were divided into the following groups: Wild-type vehicle (WT VEH) group, WT cisplatin (WT CIS) group, and B1KO cisplatin (B1KO CIS) group. For the experiments with R-715 (B1R antagonist), the animals were divided as follows: vehicle group, cisplatin-treated group, and cisplatin plus R715-treated group. We used n=6-7 for each experiment and condition.

### Cisplatin treatment

Cisplatin treatment consisted of repeated administration of cisplatin at a dose of 7 mg/kg (Bergamo, Brazil) intraperitoneally (*ip*) once a week for four weeks. The mice were euthanized 30 days after the last cisplatin injection. The vehicle groups were treated exactly the same way, and received 0.9% NaCl instead of cisplatin, in the same volume as the treated groups.

### B1R antagonist

R-715 (Sigma-Aldrich, USA) was used as a B1R antagonist. It was injected *ip* 48, 24, and 1 h prior to each cisplatin injection, and 24 h after each cisplatin injection at a dose of 800 µg/kg (15,17).

### Blood sampling and tissue collection

The mice were anesthetized with ketamine (91 mg/kg) and xylazine (9.1 mg/kg) *ip* and blood was collected via cardiac puncture. For serum collection, blood samples were allowed to clot for 2 h at room temperature and then centrifuged for 20 min at 2000 *g* at 4°C. The samples were then stored at -20°C. Kidney tissue was collected and the renal capsule was removed. Transversal cuts were performed and the kidneys were immediately frozen in nitrogen and then stored at -80°C.

### Renal function

Serum creatinine and urea levels were used to determine renal function. Samples were analyzed using commercially available colorimetric assay kits (Labtest, Brazil). The mice were housed individually and urine was collected in metabolic cages over 24 h one day prior to euthanasia. Protein concentration was determined using the Sensiprot assay kit (Labtest).

### Histological analysis

The kidneys were fixed in 10% formaldehyde and then dehydrated and embedded in paraffin. Sections (4 µm) were cut and stained with hematoxylin eosin and Sirius Red. At least six subcortical fields were visualized and analyzed for each mouse using a Leica DM4000 microscope (Germany) at a 200× magnification. The tubular injury score was determined based on the percentage of tubules showing luminal casts, cell detachment or dilation, and assigned according to the following scale: 0) 0-5%; 1) 6-25%; 2) 26-50%; 3) 51-75%; and 4) >75%. Histological analysis was performed blind to the experimental groups to assess tubulointerstitial fibrosis based on the Sirius Red-positive area, and the score was assigned according to the following scale: 1) ≤25%; 2) 26 to 50%; 3) 51 to 75%; and 4) >75%.

### Gene expression

Kidney samples were frozen at −80°C immediately after collection. Total RNA was isolated using TRIzol Reagent (Invitrogen, USA). RNA integrity was assessed by electrophoresis on an agarose gel. RNA was measured using Nanodrop 2000 (Thermo Scientific, USA), and all samples were standardized to 1000 ng for the cDNA synthesis. Then, cDNA was synthesized using the “High-Capacity cDNA Reverse Transcription kit” (Applied Biosystems, USA). Standard curves were plotted to determine the amplification efficiency for each primer pair and melting curve analyses were performed to prove, for each reaction, that each set of primers never amplified primer-dimers and that the products of each reaction were single amplicons. Real-time PCR was performed using the SYBR Green system (Thermo Scientific) with specific primers for β-actin, 18S, collagen 1A1, α-SMA, TGF-β, fibronectin, collagen 3, collage 4, vimentin, TNF-α, NGAL, KIM-1, endothelin-1, arginase-1, CD206, IL-4R, YM1, iNOS, and MMP-9. The primers were designed using Primer3web (https://primer3.ut.ee/) and their specificity was confirmed using NCBI primer-BLAST (NCBI/NLM, USA). Their sequences are listed in Table 1. The cycling conditions were as follows: 10 min at 95°C, followed by 45 cycles of 30 s at 95°C, 30 s at 60°C, and 30 s at 72°C. Target mRNA expression was normalized to β-actin and 18 s and reported as a relative value using the comparative threshold cycle method (18). The expression levels of the genes of interest were normalized to the control group and reported as fold change.

**Table 1:** Sequences of the primers used for real-time PCR assays.

Gene	Forward 5′-3′	Reverse 5′-3′	Amplicon (number of base pairs)
18S	CGC CGC TAG AGG TGA AAT TC	TCT TGG CAA ATG CTT TCG C	64
β-actin	CTG GCC TCA CTG TCC ACC TT	CGG ACT CAT CGT ACT CCT GCT T	61
NGAL	ATG TGC AAG TGG CCA CCA CG	CGC ATC CCA GTC AGC CAC AC	249
TNF-α	GCC TCT TCT CAT TCC TGC TTG	CTG ATG AGA GGG AGG CCA TT	115
KIM-1	TGT CGA GTG GAG ATT CCT GGA TGG T	GGT CTT CCT GTA GCT GTG GGC C	128
TGF-β1	CAA CAA TTC CTG GCG TTA CCT TGG	GAA AGC CCT GTA TTC CGT CTC CTT	128
Col3	TGG ACC AAA AGG TGA TGC T	CAA GAC CTC GTG CTC CAG T	116
Col4	TCC CTG GTA GTC GTG GAG A	GCC TGC TTG TCC TTT TTC A	86
Vimentin	CAG GAG GAG ATG CTC CAG A	AGG TCA AGA CGT GCC AGA G	92
Endothelin 1	GCC ACA GAC CAG GCA GTT AG	CGA AAA GAT GCC TTG ATG CTA TT	239
iNOS	CTG CTG GTG GTG ACA AGC ACA TTT	ATG TCA TGA GCA AAG GCG CAG AAC	167
MMP-9	ACG GAC CCG AAG CGG ACA TT	TTG CCC AGC GAC CAC AAC TC	163
Arginase-1	CGC CTT TCT CAA AAG GAC AG	CCA GCT CTT CAT TGG CTT TC	204
CD206	CAA GGA AGG TTG GCA TTT GT	CCT TTC AGT CCT TTG CAA GC	111
IL-4R	CAC AGT GCA CGA AAG CTG AA	ATG GGC ACA AGC TGT GGT AG	157
YM1	CCC CTG GAC ATG GAT GAC TT	AGC TCC TCT CAA TAA GGG CC	125
Collagen1A1	CCC CGG GAC TCC TGG ACT T	GCT CCG ACA CGC CCT CTC TC	180
α-SMA	TTG GAA AAG ATC TGG CAC CAC	GCA GTA GTC ACG AAG GAA TAG	368
Fibronectin	CCT ACG GCC ACT GTG TCA CC	AGT CTG GGT CAC GGC TGT CT	140

### ELISA assay

Kidney samples were frozen and stored at −80°C immediately after collection. Renal YM1 (mouse chitinase-3-like protein 3, MBS288492) levels were quantified using ELISA mouse kit specific for the analyte (MyBioSource Inc, USA), according to the manufacturer's instructions.

### Statistical analyses

All data are reported as means±SE. The significance of intergroup differences was assessed by one-way analysis of variance (ANOVA) with the Tukey's correction for multiple comparisons. Comparisons between two groups were conducted using the two-tailed *t*-test when the data were normally distributed. Statistical significance was established at P<0.05. All statistical analyses were performed using GraphPad Prism 8 (GraphPad, USA).

## Results

### Kinin B1 receptor deletion worsened chronic renal dysfunction and structural injury induced by multiple cisplatin treatment

**Figure 1:**
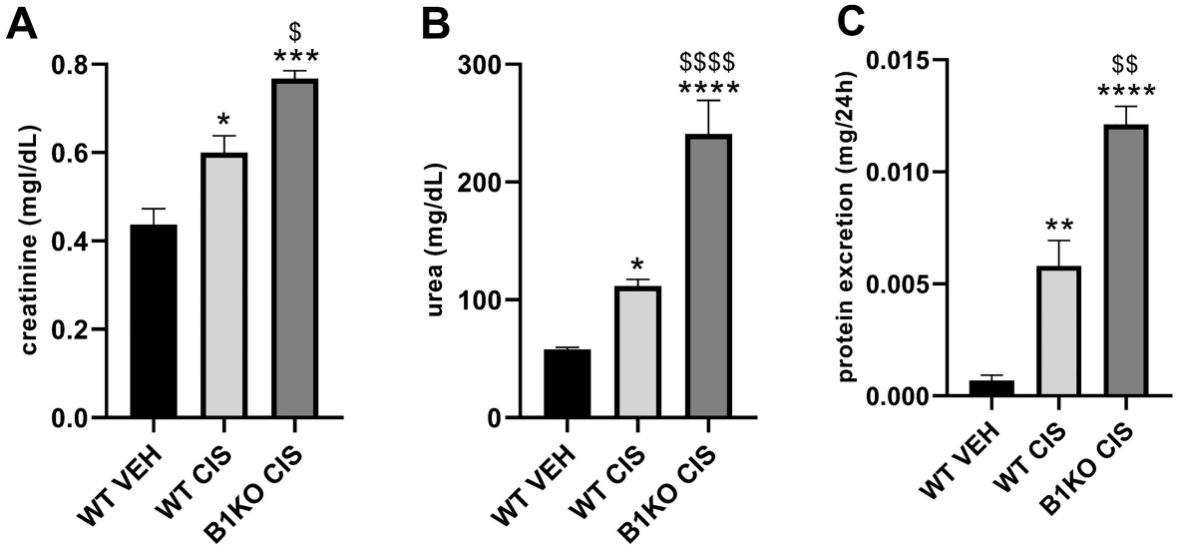
Kinin B1 receptor deficiency worsens cisplatin-induced renal dysfunction. Serum creatinine (**A**), urea (**B**), and protein excretion (**C**). Data are reported as means±SE (n=4-5 per group). *P<0.05, **P<0.01, ***P<0.001, ****P<0.0001 compared to the WT (wild type) VEH (vehicle) group; ^$^P<0.05, ^$$^P<0.01, ^$$$$^P<0.0001 compared to the WT CIS (cisplatin) group (one-way ANOVA followed by *post hoc* Tukey's test).

Mice were treated with cisplatin (7 mg/kg, *ip*) once a week for four consecutive weeks and then followed-up for thirty days. Repeated cisplatin treatment led to increased serum creatinine and urea and increased urinary protein levels, and kinin B1 receptor knockout (B1KO) mice treated with cisplatin showed an exacerbation in these three parameters (Figure 1).

**Figure 2:**
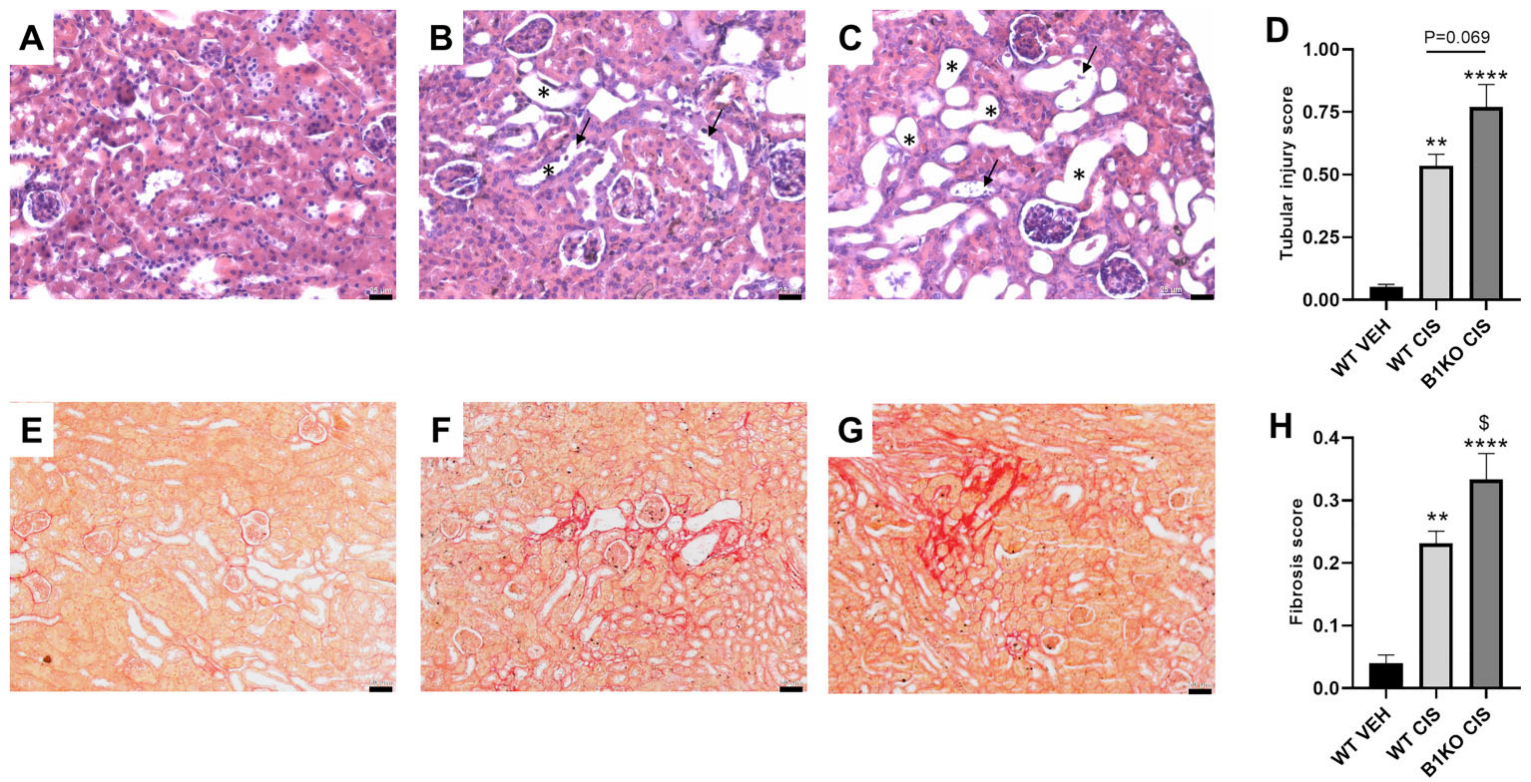
Kinin B1 receptor deficiency worsens cisplatin-induced tubular injury and renal fibrosis. Hematoxylin and eosin staining, representative images of wild-type vehicle (WT VEH) (**A**), WT CIS (cisplatin) (**B**), and B1KO CIS (**C**) groups. Asterisks indicate tubular dilation and arrows indicate tubular cell detachment. Scale bars, 25 µm. Tubular injury score (**D**) was determined based on the percentage of tubules showing luminal casts, cell detachment, or dilation. Sirius red-stained representative images: WT VEH (**E**), WT CIS (**F**), and B1KO CIS (**G**) groups. Scale bars= 50 µm. Fibrosis score was determined by Sirius red positive areas (**H**). Data are reported as means±SE (n=4-5 per group). **P<0.01, ****P<0.0001 compared to the WT VEH group; ^$^P<0.05 compared to the WT CIS group (one-way ANOVA followed by *post hoc* Tukey's test).

Cisplatin treatment led to tubular injury and B1 receptor deletion enhanced this response, as evidenced by the tubular injury score (Figure 2A-D). Moreover, renal fibrosis, which is increased after repeated cisplatin treatment, was also exacerbated in B1KO mice when compared to cisplatin-treated WT mice (Figure 2E-H).

### Kinin B1 receptor antagonism enhanced chronic renal dysfunction and tubular injury induced by multiple cisplatin treatment

**Figure 3:**
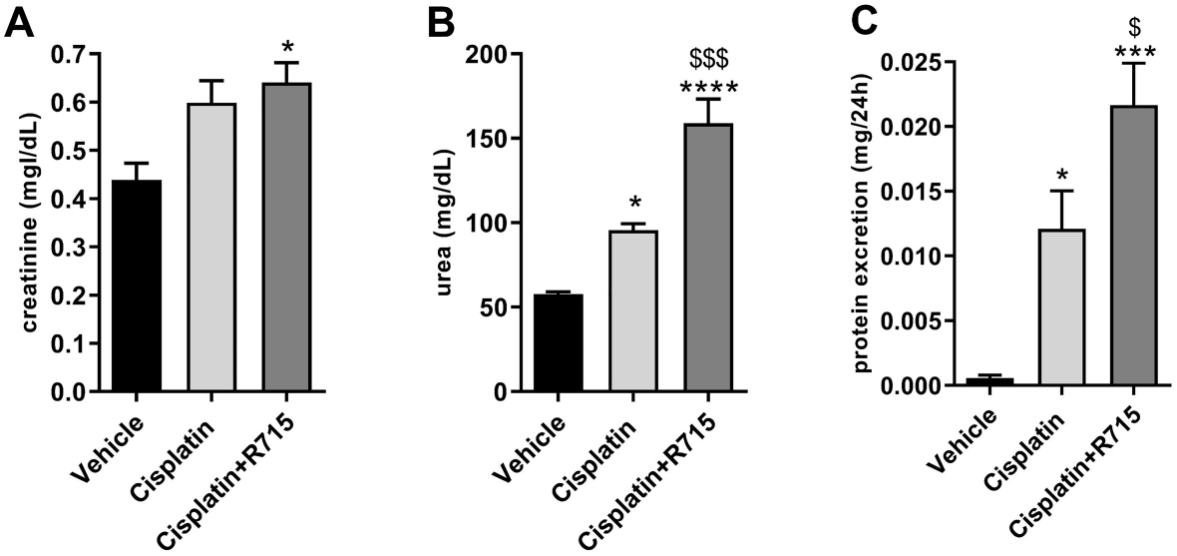
Kinin B1 receptor antagonism worsens cisplatin-induced renal dysfunction. Serum creatinine (**A**), urea (**B**), and protein excretion (**C**). Data are reported as means±SE (n=6 per group). *P<0.05, ***P<0.001, ****P<0.0001 compared to the vehicle group; ^$^P<0.05, ^$$$^P<0.001 compared to the cisplatin group (one-way ANOVA followed by *post hoc* Tukey's test).

Repeated cisplatin treatment did not significantly increase serum creatinine levels, whereas B1R antagonism with the administration of R-715 and cisplatin led to increased serum creatinine levels (Figure 3A). Multiple doses of cisplatin increased urea levels and proteinuria, and R-715 treatment exacerbated both parameters (Figure 3B and C).

**Figure 4:**
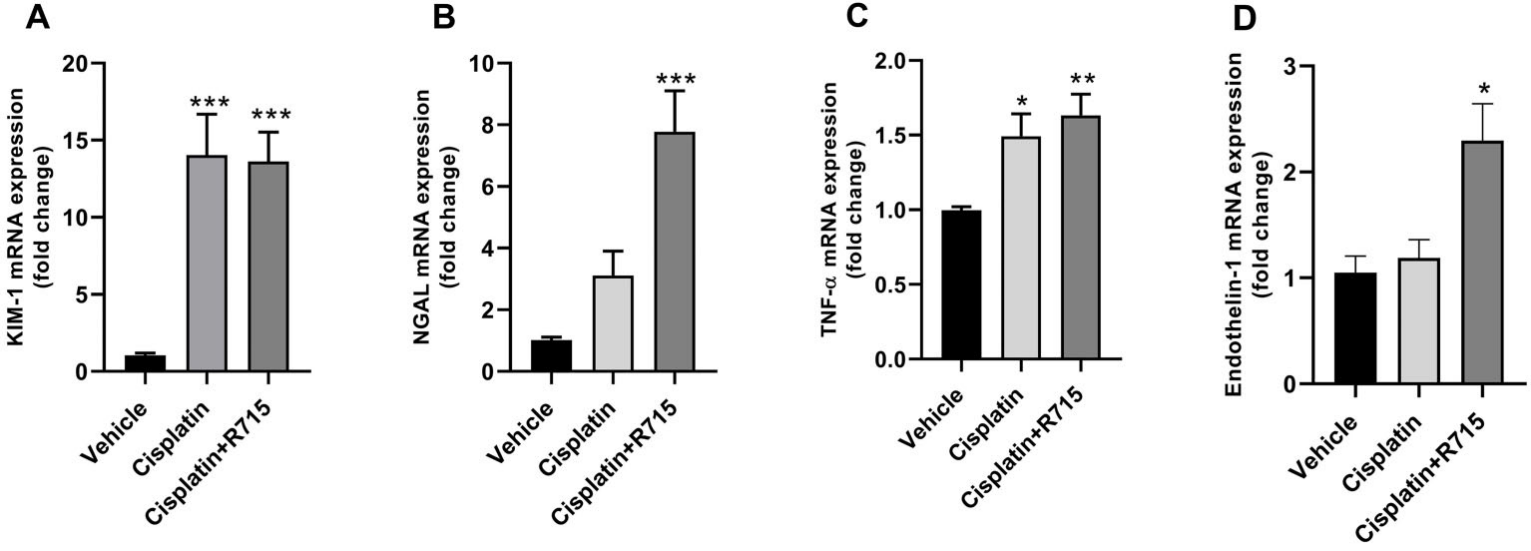
Multiple cisplatin treatment induces kidney damage. Renal mRNA levels of KIM-1 (**A**), NGAL (**B**), TNF-α (**C**), and endothelin-1 (**D**). Data are reported as means±SE (n=6 per group). *P<0.05, **P<0.01, ***P<0.001 compared to the vehicle group (one-way ANOVA followed by *post hoc* Tukey's test).

Then, qPCR was performed for three biomarkers: kidney injury molecule-1 (KIM-1), a biomarker of renal tubular cells injury; neutrophil gelatinase-associated lipocalin (NGAL), which is highly produced and released from tubular cells after renal damage and is a reliable marker for the severity of kidney damage; and tumor necrosis factor-alpha (TNF-α), a pro-inflammatory cytokine that has an important role on cisplatin-induced renal injury. Cisplatin treatment increased mRNA expression of KIM-1 and TNF-α in renal tissue (Figure 4A and C), whereas R-715-treated mice showed exacerbated NGAL levels (Supplementary Figure S1). R-715 and cisplatin induced an increase in NGAL and endothelin-1 expression (Figure 4B and D).

**Figure 5:**
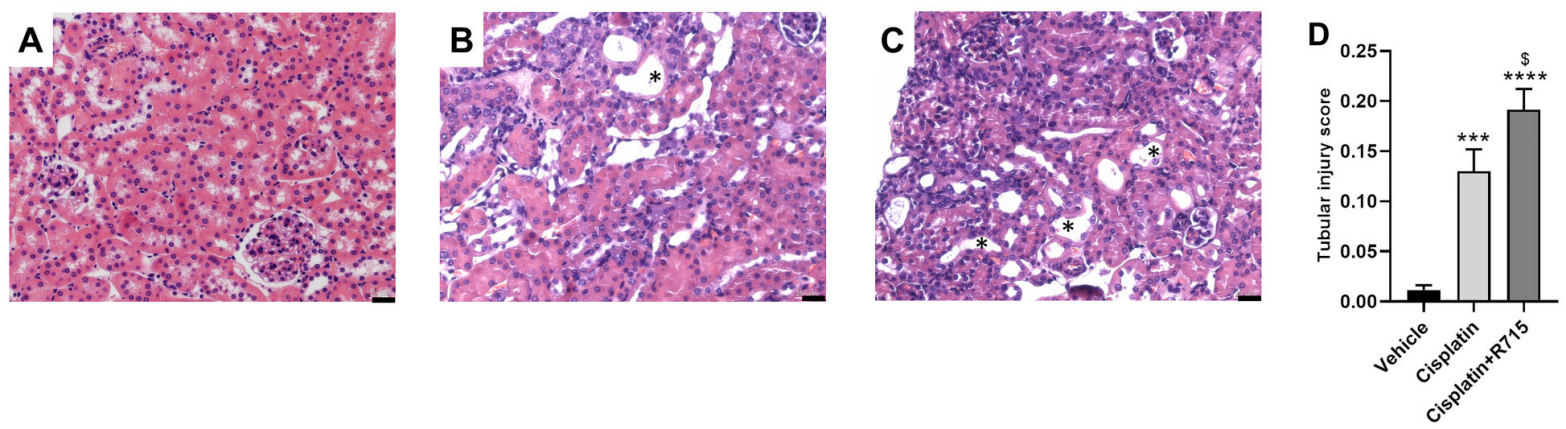
Kinin B1 receptor antagonism worsens cisplatin-induced tubular injury. Hematoxylin and eosin staining, representative images of vehicle (**A**), cisplatin (**B**), and cisplatin+R715 (**C**) groups. Asterisks indicate tubular dilation. Scale bars, 25 µm. Tubular injury score (**D**) was determined based on the percentage of tubules showing luminal casts, cell detachment, or dilation. Data are reported as means±SE (n=6 per group). ***P<0.001, ****P<0.0001 compared to the vehicle group; ^$^P<0.05 compared to the cisplatin group (one-way ANOVA followed by *post hoc* Tukey's test).

Histological analysis confirmed that tubular injury was exacerbated in the R-715 group compared to mice receiving cisplatin only (Figure 5A-D).

### Kinin B1 receptor antagonism exacerbated tubulointerstitial fibrosis

**Figure 6:**
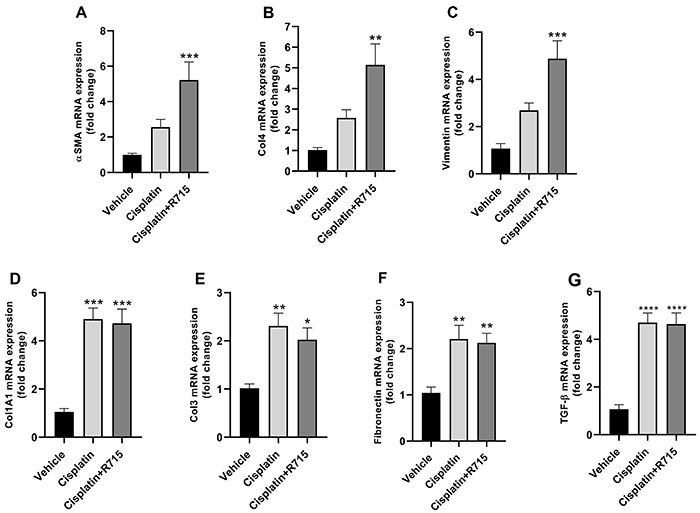
Multiple cisplatin treatment increases renal fibrosis markers. Kidney mRNA levels of α-SMA (**A**), collagen 4 (**B**), vimentin (**C**), collagen 1A1 (**D**), collagen 3 (**E**), fibronectin (**F**), and TGF-β (**G**). Data are reported as means±SE (n=6 per group). *P<0.05, **P<0.01, ***P<0.001 ****P<0.0001 compared to the vehicle group (one-way ANOVA followed by *post hoc* Tukey's test).

**Figure 7:**
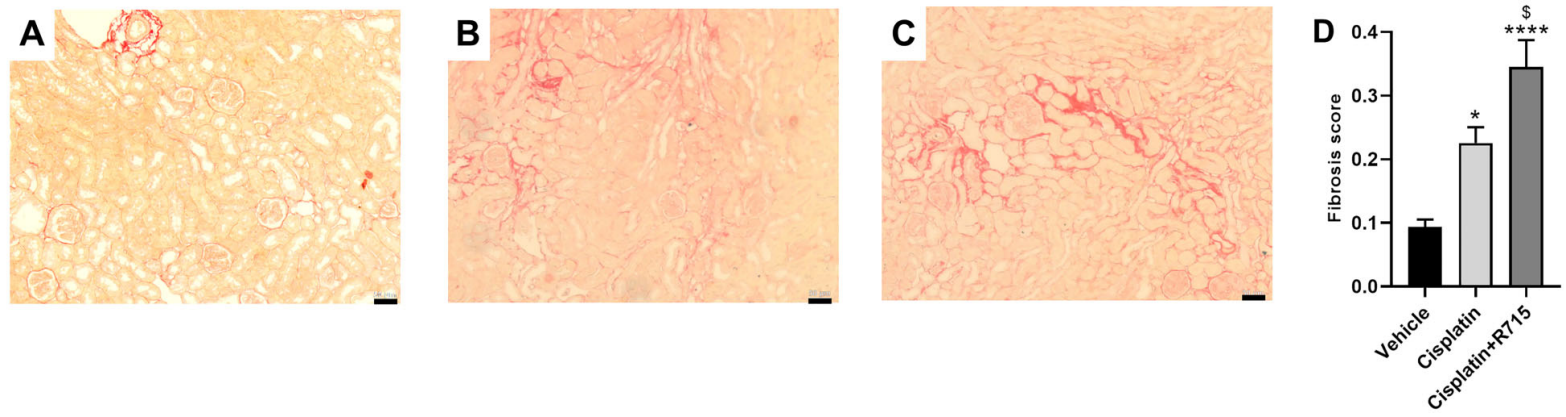
Kinin B1 receptor antagonism exacerbates cisplatin-induced tubulointerstitial fibrosis. Sirius red stained representative images of the vehicle (**A**), cisplatin (**B**), and cisplatin+R715 (**C**) groups. Scale bars, 50 µm. Fibrosis score was determined by Sirius red positive areas (**D**). Data are reported as means±SE (n=6 per group). *P<0.05, ****P<0.0001 compared to the control group; ^$^P<0.05 compared to the cisplatin group (one-way ANOVA followed by *post hoc* Tukey's test).

Multiple cisplatin doses showed a non-statistical increase of renal mRNA expression of fibrotic markers, such as alpha smooth muscle actin (α-SMA), collagen 1A1, collagen 3, collagen 4, fibronectin, transforming growth factor beta (TGF-β), and vimentin (Figure 6A-G). Treatment with R715 exacerbated α-SMA, collagen 4, and vimentin mRNA levels in renal tissue compared to the cisplatin-treated group (Supplementary Figure S1), whereas the other markers showed an increase similar to the cisplatin groups (Figure 6D-G). Fibrosis was confirmed in the kidneys of cisplatin-treated mice by Sirius red staining, and kinin B1R antagonism increased the fibrosis score (Figure 7A-D).

### Kinin B1 receptor antagonism impaired M2 macrophages response

**Figure 8:**
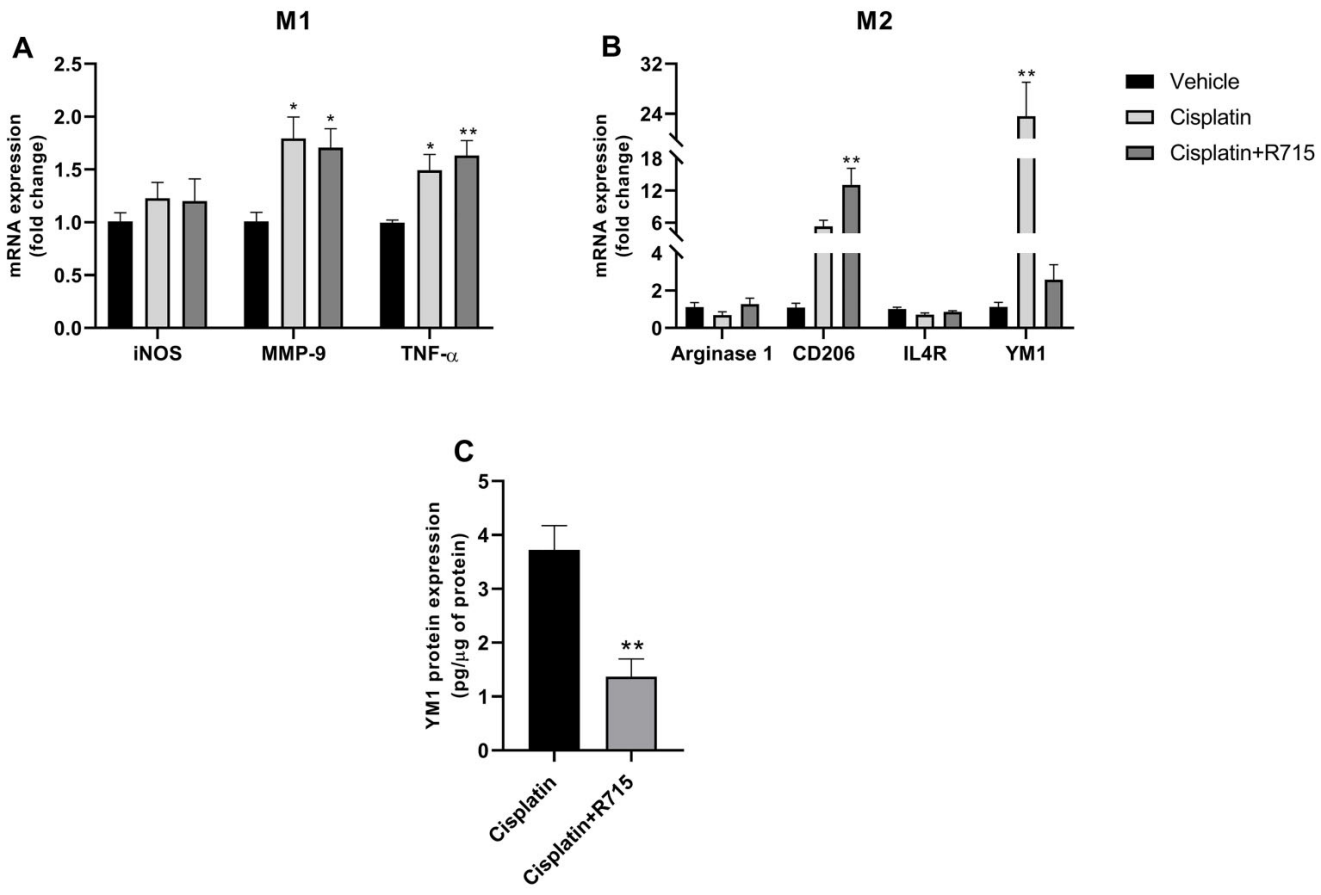
Kinin B1 receptor antagonism did not increase YM1 expression. Renal mRNA expression of M1 markers (**A**), M2 markers (**B**), and renal YM1 (**C**) protein levels. Data are reported as means±SE (n=6 per group). *P<0.05, **P<0.01 compared to the vehicle group (one-way ANOVA followed by *post hoc* Tukey's test).

Macrophages play an important role in wound repair processes, in which the profile change from M1 pro-inflammatory macrophages to M2 anti-inflammatory and wound-healing macrophages has shown to be an important process in the development of renal fibrosis. In this study, some M1-polarization markers were found to be increased after cisplatin exposure, either alone or in association with R-715 (Figure 8A). As for M2 markers, YM1 mRNA expression was increased in the cisplatin group (Figure 8B and Supplementary Figure S1), whereas its levels were not increased in R-715-treated mice. These results were confirmed with protein expression by ELISA assay (Figure 8C).

## Discussion

Cisplatin is a well-known and efficient chemotherapeutic drug used against several solid tumors, including testes, bladder, lung, ovarian, and head and neck cancers. It has a high rate of success against cancer; however, toxicity limits its use (7,8). Several toxicities have been reported with the use of cisplatin, and nephrotoxicity is the most documented one, affecting about 30% of patients undergoing cisplatin treatment (7,8). The long-term effects of cisplatin on the kidneys have been recently investigated and indicate that AKI can develop or progress to CKD (19). We have previously shown that the deletion and antagonism of kinin B1 receptor (which is highly up-regulated under inflammatory conditions) can mitigate cisplatin-induced AKI (15).

Chronic treatment with cisplatin led to renal dysfunction, as evidenced by the increased levels of serum urea and proteinuria, and surprisingly, the kinin B1R antagonist, R-715, in addition to not protecting against cisplatin, also exacerbated cisplatin-induced renal dysfunction.

Furthermore, we observed that cisplatin induced increased renal expression of tubular cell injury markers, represented by KIM-1, NGAL, and TNF-α, which was confirmed by histological analyses. Kinin B1R antagonist exacerbates NGAL expression, a marker of tubular cell injury and an important indicator of the severity of kidney damage (20,21), which was confirmed by histological analyses in our study, showing enhanced tubular injury in mice with B1R inhibition.

Indeed, cells of proximal tubules are the most affected by cisplatin acute toxicity, and a maladaptive repair or repeated insults in these cells lead to CKD (3). Additionally, AKI leads to increased fibrogenesis, which is a risk factor for CKD progression (5). In this study, cisplatin treatment led to increased gene expression of fibrotic markers in renal tissue, in which treatment with R-715 increased renal gene expression of some important markers. Interstitial fibroblasts, myofibroblasts, tubules, and inflammatory cells have been reported to be the main sources of increased matrix protein production in renal fibrosis (3,6,22).

Unlike what was observed in AKI, kinin B1 receptor antagonism did not promote protection against CKD; in fact, it exacerbated renal injury. In our previous study, we found that B1R deletion decreases immune cell migration after cisplatin acute toxicity (15). Macrophages, neutrophils, dendritic cells, and natural killer cells were increased after cisplatin exposure, while this phenomenon was not observed in B1R-null mice (15). After injury, macrophages can adopt multiple phenotypes, including M1 and M2. M1 are considered pro-inflammatory macrophages, and produce cytokines such as IL-1, IL-6, and TNF-α, whereas M2 are considered anti-inflammatory macrophages, and produce mannose receptor, IL-10, and IL-4 (23,24). It has been shown that YM1 acts directly as a repair molecule, by regulating the balance of type 2 T helper, which is important to prevent fibrosis (25). The pro-repair actions of YM1 are related to its ability to bind to ECM components (26,27) and regulate the availability of reparative proteins (28). YM1 blockade prevented efficient lung repair (25). The expression of YM1 was found to be increased in the treatment with cisplatin alone, but when associated with B1 receptor antagonist, neither YM1 induction, nor gene or protein expression was observed, showing that the antagonism of B1 receptor led to a deficient renal repair process mediated by YM1. The important role of CD206 in switching M1/M2 profiles is observed in the wound healing process (29,30). However, some studies show that, chronically, CD206 is up-regulated and closely related to fibrosis (31,32), corroborating our findings that cisplatin increased CD206 expression and that the association of cisplatin with R715 exacerbated its expression. Moreover, we found no difference in the expression of arginase-1 and IL-4R, which may be due to the time of analysis.

Endothelin-1 is released by endothelial cells, epithelial cells, and mesenchymal cells. Moreover, in fibrotic state, it is also secreted by inflammatory cells, such as macrophages and neutrophils, in addition to fibroblasts and myofibroblasts (33,34). It is well documented that endothelin-1 is an important contributor to tissue fibrosis (35). In this study, we confirmed that treatment with R-715 up-regulated endothelin-1, accompanied by increased renal fibrosis. Some studies show that the polarization of M1 to M2 phenotype plays an important role in the wound-healing process in renal diseases (36,37). We believe that, since macrophages were not increased with the deletion and antagonism of B1R, polarization from M1 to M2 cannot occur, and although there is acute protection against it, some maladaptive tubular regeneration and even mild toxicity can be established and lead to renal fibrosis exacerbation. It is worth mentioning that other mediators may be involved in this more intense inflammation mechanism, since the inflammasome, the renin-angiotensin system, and the gut microbiota play an important role in triggering inflammation in both AKI and CKD (38-40).

Our findings showed that kinin B1 receptor antagonism exacerbated the damage caused by multiple cisplatin treatment. Further studies are required to better understand the mechanisms underlying the role of kinins in CKD and the specific cell types in which B1R is important for renal fibrosis. Nonetheless, we have addressed an important point, showing that kinin B1 receptor antagonism should not be used in conjunction with chronic cisplatin therapy.

## Supplementary Material

Click here to view [pdf].
